# Reconsidering political occupational therapy: implications of martial law on occupational justice in South Korea

**DOI:** 10.3389/fsoc.2025.1594638

**Published:** 2026-01-05

**Authors:** Hyub Kim

**Affiliations:** Department of Occupational Therapy, Far East University, Eumseong County, Republic of Korea

**Keywords:** human rights, political occupational therapy, occupational therapy, occupational justice, martial law

## Abstract

**Introduction:**

The imposition of martial law on December 3, 2024, in South Korea has reignited critical discussions on the intersection of political systems and occupational justice. Authoritarian governance imposes significant restrictions and disruption of meaningful occupations and exacerbating inequities, particularly among marginalized communities. This study examines the implications of martial law through the lens of occupational justice and political occupational therapy. Additionally, the introduction engages with organizational communication theories, such as institutional discourse, public rhetoric, and crisis communication frameworks, to position occupational therapists as communicative agents of systemic change. By integrating historical events such as the Gwangju Uprising, the study aims to highlights systemic barriers to occupational engagement and the broader socio-political determinants of occupation.

**Methods:**

This study employs an exploratory review methodology to analyze the impact of martial law on occupational justice. Drawing from historical, political, and occupational therapy literature, the review synthesizes theoretical frameworks, policy analyses, and case studies to examine how authoritarian governance shapes occupational participation. The study also explores the role of occupational therapists in addressing socio-political determinants of occupation during periods of political crisis.

**Results:**

Findings indicate that martial law imposes systemic occupational disruptions, disproportionately affecting marginalized communities, including political dissidents, low-income workers, and individuals with disabilities. Restrictions on movement, expression, and access to essential services limit occupational engagement, reinforcing structural inequalities. Historical comparisons with events such as the Gwangju Uprising demonstrate recurring patterns of occupational injustice under authoritarian regimes. The analysis suggests that occupational therapy, when positioned as an inherently political discipline, has the potential to advocate for occupational rights and mitigate the impact of governance-related disruptions.

**Conclusion:**

This study argues for a paradigm shift in occupational therapy, urging practitioners to transcend traditional clinical roles and engage in policy advocacy. Recognizing occupational justice as a fundamental human right, occupational therapists can play a critical role in addressing socio-political determinants of occupation. By advocating for policies that safeguard occupational engagement during crises, the profession can contribute to a more just and equitable society.

## 1 Introduction

On December 3, 2024, the imposition of martial law by South Korean President Yoon Suk Yeol (Lim et al., 2024) reignited debates about the devastating impact of authoritarian governance on daily life and community participation (Cooley, 2015; Heberer, 2009; Hooghe and Quintelier, 2014; Swyngedouw, 2000). This stark political event highlights the urgency of understanding how political systems shape the fundamental human right to meaningful occupation (Hammell, 2008; Hammell, 2015; Landman, 2005). The growing significance of political contexts in occupational therapy is evident in addressing occupational justice challenges, as demonstrated by the effects of apartheid policies on participation in South Africa (Gradín, 2019; Kronenberg, 2018; Watson, 2015) and the impact of forced migration policies on displaced populations (Fabianek et al., 2023; Mirza, 2012; Trimboli et al., 2024).

Political occupational therapy, a growing area of inquiry, examines the ways in which socio-political systems influence individuals’ and communities’ occupational participation. It refers to the intentional engagement of occupational therapy practices and principles to address systemic political and social issues that impede occupational justice (Townsend and Wilcock, 2004). For example, in apartheid-era South Africa, occupational therapy played a role in empowering marginalized communities through community-based programs that sought to restore agency and participation (Duncan et al., 2005). Similarly, in refugee camps, occupational therapists have addressed the occupational deprivation caused by forced displacement, promoting resilience and self-reliance among displaced populations (Siddiqui et al., 2019). It encompasses the analysis of how power dynamics, policies, and governance structures shape occupational opportunities and limitations, such as the effects of authoritarian regimes on community engagement or the role of activism in preserving occupational rights (Hammell and Iwama, 2012). In South Korea, the imposition of martial law—such as during the Gwangju Uprising in 1980, when military rule suppressed civil rights and led to widespread violence—poses significant threats to the fundamental principles of occupational justice, including autonomy, equity, and participation (Shin, 2020).

Many cultures approach the open discussion of politics with great caution. However, politics is inherently a human occupation and considering the simultaneous emergence of inequalities alongside the progression of civilization, both occupation and science possess political dimensions (Kronenberg and Pollard, 2005). This concept of political occupational therapy expands the traditional scope of occupational therapy by recognizing that occupations are deeply influenced by the political and social structures in which individuals and communities operate. According to Kronenberg (2018), political occupational therapy emphasizes advocacy, policy influence, and systemic change to ensure equitable access to meaningful occupations for all individuals.

Examples of political occupational therapy include efforts to design community programs for refugees that counteract the occupational deprivation caused by forced migration policies, or initiatives that promote participatory urban planning to combat occupational injustices in marginalized communities. Another example is the development of culturally sensitive occupational frameworks that challenge and dismantle discriminatory practices affecting Indigenous populations.

In the context of martial law in South Korea, political occupational therapy could involve advocating for policies that protect citizens’ rights to participate in daily activities, fostering community resilience through collective occupations, and collaborating with policymakers to mitigate the negative impacts of authoritarian governance on occupational participation. These practices highlight the potential of occupational therapy to act as a transformative force in addressing not only individual needs but also structural barriers to occupational justice (Hammell, 2017).

The inclusion of political occupational therapy within this discussion underscores the need for the profession to evolve in response to complex societal challenges. By integrating advocacy and systemic change into practice, occupational therapy can further its commitment to promoting justice and human rights (Kim, 2024b).

Martial law disrupts everyday life through censorship, restricted mobility, and the suppression of dissenting voices, thereby creating occupational injustices that disproportionately affect marginalized and vulnerable groups, including women, children, and ethnic minorities. For example, women often faced increased surveillance and restrictions on social roles, children’s access to education and safe play environments were severely limited, and ethnic minorities experienced intensified discrimination and exclusion from civic participation (Jurado, 1980; Oh, 2020). These occupational injustices highlight broader societal dysfunctions. For instance, the suppression of labor unions and student protests during periods of martial law in South Korea not only restricted individual freedoms but also curtailed collective occupational engagement and advocacy (Kang, 2024). These restrictions disrupted critical societal functions, including education and employment sectors, by stifling academic discourse, hindering workforce mobilization, and limiting access to safe and fair labor conditions (Kang, 2024). Despite the potential for occupational therapy to address these challenges, there remains a lack of critical engagement with the implications of martial law within the field. This may stem from political barriers, such as the risks associated with criticizing authoritative governance, and academic barriers, including a traditional focus on individual and clinical aspects of occupational therapy rather than systemic or political issues (Baek, 2024). Addressing these gaps requires an interdisciplinary approach that considers the structural determinants of occupation.

This paper aims to critically review the concept of political occupational therapy in the context of South Korea’s martial law, a setting where the intersection of governance and occupation vividly demonstrates the impact of systemic oppression on daily life and community participation. It explores the systemic barriers imposed by martial law, such as the suppression of free speech, restricted access to education, and limitations on community organization, and how these directly undermine the principles of occupational justice. The analysis highlights the limitations of existing occupational therapy literature in addressing political oppression and examines the potential for occupational therapists to act as agents of social change by challenging these systemic inequities. By evaluating current frameworks, such as those addressing occupational justice and community-based interventions under oppressive political systems, including critical occupational therapy and liberation theory, and proposing avenues for future research, this review seeks to contribute to the broader discourse on the socio-political dimensions of occupation and the evolving role of occupational therapy in fostering justice and equity. Furthermore, this discussion is situated within the broader field of occupational science, drawing on scholarship that examines the socio-political determinants of occupation and the role of occupational scientists in advancing justice-oriented practice (Hocking, 2019; Galvaan, 2015; Wilcock and Hocking, 2024). This expansion acknowledges that while occupational therapy practice is central to addressing these issues, the interdisciplinary contributions of occupational science professionals are equally vital for understanding and responding to systemic occupational injustices.

## 2 Methods

### 2.1 Study design

This study employs an exploratory review methodology to examine the impact of martial law on occupational justice in South Korea. An exploratory review is used to synthesize existing literature, historical events, and theoretical frameworks to generate new insights into complex and underexplored topics. This approach is particularly suited for analyzing the socio-political determinants of occupation within an occupational therapy framework.

### 2.2 Data sources and selection

Keywords used included “martial law,” “occupational justice,” “political occupational therapy,” “South Korea,” “human rights,” and “authoritarian governance.” Eligibility criteria included peer-reviewed sources policy documents and credible historical accounts directly related to martial law and occupational justice.

The review draws from a range of sources, including peer-reviewed journal articles, policy documents, historical records, and reports from human rights organizations. Key literature on occupational justice, political occupational therapy, and authoritarian governance was identified through academic databases such as PubMed, Scopus, and Google Scholar. Historical analysis focused on South Korea’s past instances of martial law, including the Gwangju Uprising, to provide a comparative perspective on recurring patterns of occupational disruption.

### 2.3 Analytical approach

The Framework of Occupational Justice (Townsend, 2012) guided the thematic analysis, providing a structured lens to assess participation restrictions, systemic inequities, and access to meaningful occupation.

A thematic analysis was conducted to identify key patterns in how martial law influences occupational engagement. The review examined structural barriers to occupation, disparities in occupational participation among marginalized groups, and the potential role of occupational therapy in addressing these challenges. Literature was analyzed using the occupational justice framework, emphasizing access to meaningful occupation, participation restrictions, and systemic inequities.

## 3 Results

Martial law is a legal framework that temporarily transfers authority from civilian governance to military control in national emergencies when civil authorities are regarded being unable to function (Encyclopaedia Britannica, 2025). For instance, during the 1981 martial law in Poland, the government, under General Wojciech Jaruzelski, suspended political freedoms, restricted movement, and suppressed solidarity movements, creating widespread occupational and social injustices (Goldman and Engel, 1982). Such examples provide a comparative lens to understand the broader implications of martial law globally. In South Korea, this governance model has historically been invoked during periods of socio-political upheaval, with notable examples including the 1980 Gwangju Uprising (Shin, 2020). This uprising began as a protest against martial law and escalated into a violent confrontation between civilians and the military, resulting in significant loss of life and widespread human rights violations (Kang, 2024; Oh, 2020). The event has since become a symbol of resistance against authoritarianism and underscores the profound occupational and social injustices that accompany such measures. These measures typically result in the suspension of civil liberties, restrictions on mobility, and censorship, creating environments that obstruct occupational participation.

The December 2024 declaration of martial law followed escalating public dissent over economic inequalities and allegations of government corruption, which culminated in mass protests and widespread strikes. These events echoed similar patterns of systemic suppression seen during earlier periods of political unrest in South Korea. These historical and contemporary examples underscore the profound occupational injustices caused by martial law, affecting autonomy, equity, and participation at individual and community levels. Given that occupational justice is a fundamental human right, these infringements constitute not only occupational but also human rights violations, reinforcing the interdependence between the two concepts. For example, during the December 2024 martial law declaration in South Korea, citizens faced restrictions on mobility that limited access to workplaces and educational institutions. Additionally, censorship curtailed the ability to engage in meaningful communication and advocacy, while community organizations experienced shutdowns, disrupting collective activities essential for social cohesion (Baek, 2024). Table 1 provides the martial law decree declared on December 3, 2024.

**Table 1:** South Korea’s martial law decree (Reuters, 2024).

No.	Declared decree
1	All political activities, including the activities of the National Assembly, local councils, and political parties, political associations, rallies and demonstrations, are prohibited.
2	All acts that deny or attempt to overthrow the liberal democratic system are prohibited, and fake news, public opinion manipulation, and false propaganda are prohibited.
3	All media and publications are subject to the control of the Martial Law Command.
4	Strikes, work stoppages and rallies that incite social chaos are prohibited.
5	All medical personnel, including trainee doctors, who are on strike or have left the medical field must return to their jobs within 48 h and work faithfully. Those who violate will be punished in accordance with the Martial Law.
6	Innocent ordinary citizens, excluding anti-state forces and other subversive forces, will be subject to measures to minimize inconvenience in their daily lives.
Violators of the above proclamation may be arrested, detained, and searched without a warrant in accordance with Article 9 of the Martial Law Act of the Republic of Korea (Special Measures Authority of the Martial Law Commander), and will be punished in accordance with Article 14 of the Martial Law Act (Penalties).	

### 3.1 Systemic barriers to occupational justice

Under martial law, social justice is compromised through systemic barriers such as censorship, restricted mobility, and suppression of dissent (Baek, 2024; Human Rights Watch, 2011; Oh, 2020’ Shin, 2020). A comparison can be drawn with Poland’s 1981 martial law, where similar restrictions were imposed, including limited movement, censorship of media, and suppression of political activists (Goldman and Engel, 1982). Similarly, the 2019 protests in Hong Kong saw widespread disruptions, with occupational participation significantly hindered by government-imposed curfews and the closure of public spaces, impacting education, employment, and community activities (Purbrick, 2019). These parallels provide a broader context to understand the occupational injustices faced under such regimes globally. A similar pattern was observed during the Hong Kong protests in 2019, where digital censorship and limited internet access disrupted grassroots advocacy and hindered the sharing of crucial information among affected communities (Purbrick, 2019). The unilateral implementation of healthcare reforms led to the resignation of numerous medical professionals, creating significant gaps in the healthcare system (Baek, 2024). Among the decrees issued under martial law, it was explicitly stated that medical professionals who did not return to work would face severe punishment. This decree highlighted the lack of communication and transparency in the government’s policymaking, which had already limited public access to healthcare, causing considerable harm to the population. Furthermore, the decree’s use of terminology typically reserved for serious crimes, such as treason, in describing the punishment for healthcare professionals—including the potential for capital sentences—sparked intense backlash from the healthcare community (National Institute of Korean Language, 2025). Restricted mobility hindered access to workplaces, healthcare services, and educational institutions, further exacerbating inequities (Baek, 2024; Human Rights Watch, 2011). In the context of South Korea’s December 2024 martial law, these barriers were not realized due to its short duration, lasting only 3 h. However, a hypothetical continuation could have led to significant occupational injustices. For instance, prolonged restrictions might have disrupted access to education, workplaces, and healthcare, mirroring challenges observed in other global contexts. Experiencing 16 more martial laws in modern history, even the brief imposition of martial law created a climate of fear and uncertainty, highlighting how even temporary authoritarian measures can influence trust, civic engagement, and community cohesion. Table 2 describes most notable martial laws imposed in South Korea since its establishment in 1948.

**Table 2:** Most notable imposition of martial law in South Korea.

Year	Incidence
1948	President Syngman Rhee declared martial law to suppress the Yeosu-Suncheon Rebellion
1960	Martial law was declared during the April Revolution to quell mass protests against electoral fraud and authoritarianism
1961	General Park Chung-hee imposed martial law following a military coup, leading to his rise to power
1972	President Park Chung-hee declared martial law to implement the Yushin Constitution, extending his authoritarian rule.
1979	Martial law was declared after the assassination of President Park Chung-hee, leading to significant political turmoil
1980	General Chun Doo-hwan expanded martial law nationwide, resulting in the Gwangju Uprising, a pro-democracy movement met with violent military suppression

Education systems, in global contexts of prolonged martial law, often face severe disruptions. Institutions are shut down or heavily regulated, depriving students of consistent learning environments, peer interactions, and extracurricular activities (Oh, 2020). Employment sectors can stagnate as workforce mobilization is suppressed, affecting economic stability and occupational engagement (Shvets et al., 2024). While such systemic effects were not directly observed in South Korea’s brief martial law, they underscore the potential occupational consequences of governance shifts if sustained over time. Reflecting on these scenarios provides valuable insights for preventive and restorative occupational therapy interventions in politically volatile settings.

### 3.2 Gaps in occupational science and occupational therapy literature

Despite the relevance of occupational therapy in addressing socio-political challenges, existing literature often neglects the broader socio-political dimensions of occupation (Duncan et al., 2005; Kronenberg and Pollard, 2005). Occupational science and therapy have traditionally focused on individual-level interventions to restore or enhance occupational engagement, often without adequately addressing the structural and systemic factors that inhibit participation. For instance, there is limited exploration of how occupational therapy can navigate challenges such as government-imposed curfews, restricted freedom of movement, or the loss of cultural rituals under authoritarian regimes (Kronenberg and Pollard, 2006; Pollard and Sakellariou, 2014; Lavin, 2005). These restrictions, which profoundly affect daily life and collective occupations, have received little attention in the occupational therapy literature, leaving a significant gap in understanding how the profession can effectively intervene in such contexts.

Case studies like the interruption of community festivals in South Korea or the suppression of union activities in Poland highlight the tangible impacts of socio-political instability on occupational engagement (Kim R., 2024). These disruptions not only limit access to meaningful occupations but also erode cultural identity and social cohesion. Similarly, systemic issues such as housing inequities during political upheavals or restricted access to education in conflict zones remain underexplored (Blankvoort et al., 2018; Lavin, 2005; Simó-Algado et al., 2002). These examples underscore the importance of addressing how systemic oppression affects not only individual participation but also the broader fabric of community occupations.

Pollard and Sakellariou (2014) argue that human occupation is inherently political, as it is shaped by access to spaces, resources, and freedoms required for participation. The relative scarcity of research examining systemic barriers to occupation reflects an underdeveloped engagement with these critical dimensions. This gap is particularly evident in contexts where government policies or authoritarian practices, such as martial law or censorship, restrict mobility, communication, and cultural practices. By neglecting these dimensions, the field risks reinforcing existing occupational inequities rather than dismantling them.

Additionally, while some studies within occupational therapy and occupational science address systemic oppression and political advocacy, these efforts are uneven and often limited in scope. Research focusing on racial hierarchies (Grenier, 2020), colonial domination (Emery-Whittington and Te Maro, 2018; Fijal and Beagan, 2019; Gerlach et al., 2018; Gibson, 2020; White and Beagan, 2020), societal ableism (Gappmayer, 2021; Grenier, 2021), and socioeconomic inequalities (Hocking, 2019) is emerging but remains insufficient. For instance, keyword searches related to systemic oppression yield relatively few publications in occupational therapy and occupational science compared to the broader body of work within the field (Pooley and Beagan, 2021). This lack of focus reflects a prevailing tendency to prioritize individualized approaches over systemic analyses, limiting the field’s capacity to advocate for structural reforms and policies that could address occupational injustices at their root.

These limitations have far-reaching implications. By failing to adequately examine and address systemic oppression, the profession risks perpetuating occupational injustices experienced by marginalized populations. Without a more robust engagement with these critical issues, occupational therapy and science remain constrained in their ability to contribute to broader social transformation. Expanding research and practice to include these dimensions would allow occupational therapists to not only respond to the immediate needs of individuals but also advocate for and implement systemic changes that promote occupational justice.

### 3.3 Potential role of occupational therapy

Occupational therapists can play a transformative role in advocating for occupational justice under oppressive political systems (Bass-Haugen et al., 2005; Kronenberg and Pollard, 2006). Examples from Myanmar’s 2021 coup and Egypt’s 2011 revolution demonstrate the profession’s potential to address occupational disruptions in politically volatile environments. For example, during the martial law period in South Korea in 1980, healthcare professionals including occupational therapists collaborated with underground community groups to provide essential services and maintain educational programs despite severe restrictions (Oh, 2020). Similarly, in South Africa’s apartheid era, therapists developed community-driven initiatives to support marginalized populations through skill-building workshops and advocacy campaigns (Frank and Muriithi, 2015; Mthembu, 2021). These examples highlight how fostering community resilience through programs that enable participation despite restrictions, collaborating with human rights organizations to address systemic injustices, and leveraging global platforms to highlight the occupational implications of martial law can create meaningful impact. By aligning practice with the principles of justice, equity, and empowerment, occupational therapists can serve as agents of social change (Hammell, 2015; Picotin et al., 2021). Justice can be practically implemented through advocacy efforts for equal resource distribution, such as campaigns for improved access to public spaces for marginalized groups (Frank and Muriithi, 2015; Hammell, 2015; Picotin et al., 2021). Equity might involve tailoring rehabilitation programs to meet the cultural and socioeconomic needs of underrepresented communities, like offering language-accessible services (Van der Merwe, 2010). In politically oppressive contexts, justice is implemented through advocacy for equitable resource distribution, while equity is achieved by tailoring interventions to meet the needs of marginalized groups such as displaced refugees, ethnic minorities, or economically disadvantaged individuals (Duncan et al., 2005; Galheigo, 2011). Empowerment is fostered by engaging communities in co-designing solutions, ensuring that occupational therapy not only addresses immediate needs but also strengthens long-term resilience and agency among affected populations (Townsend et al., 2013).

When the role of occupational therapy is transferred specifically to South Korea’s current political situation, occupational therapy may be relevant in supporting mental health, promote community resilience and empowerment of individuals, influence in shaping policy and advocacy, and research and documentation on occupational impacts and culturally relevant political occupational therapy.

## 4 Discussion

### 4.1 Political occupational therapy and occupational justice

The imposition of martial law in South Korea exemplifies the interplay between political governance and occupational justice, demonstrating the multifaceted ways authoritarian regimes disrupt participation at individual, communal, and systemic levels. Although the martial law was lifted after only 2 h, it raises important questions about the hypothetical long-term effects had it remained effective, as well as the residual occupational impacts even after its quick reversal. Political occupational therapy can be defined as the practice of analyzing and addressing how political systems impact individuals’ and communities’ occupational rights and participation, with a particular focus on advocating for systemic justice and equity. If the martial law had remained effective, it could have entrenched systemic restrictions on mobility, education, and public participation, leading to prolonged occupational deprivation and deepening societal inequalities. Even after its rapid reversal, residual effects such as systemic fear, eroded trust in public institutions, and disrupted civic engagement continue to influence occupational participation and justice. These complex dynamics underscore the urgent need for occupational therapy to integrate political occupational therapy practices, addressing both immediate occupational disruptions and the longer-term psychological, social, and systemic consequences of such measures.

### 4.2 Occupational disruptions and vulnerable populations

The psychological strain on families due to curfews and mobility restrictions underscores the need for interventions that extend beyond traditional individual-focused care. This aligns closely with the broader question of how occupational therapy can rebuild trust and restore engagement after governance shifts, emphasizing the necessity for targeted approaches that address systemic disruptions and foster resilience within vulnerable populations. For example, children faced disruptions in schooling and social development due to sudden restrictions, while elderly individuals experienced increased isolation and difficulty accessing routine healthcare services. These vulnerable groups are emphasized because their occupational participation is more sensitive to systemic restrictions, as highlighted in occupational science literature on vulnerability and marginalization. They require targeted interventions, such as virtual learning programs for children and mobile healthcare services for the elderly, to mitigate the short- and long-term impacts of these disruptions. This emphasis aligns with occupational science literature that identifies children, older adults, and marginalized socio-economic groups as particularly susceptible to systemic occupational deprivation during crises (Galvaan, 2015; Hocking, 2019). The vulnerabilities stem from intersecting factors such as limited resource access, reduced autonomy, and diminished capacity to adapt to sudden policy changes.

### 4.3 Structural barriers and the role of occupational therapy

Occupational therapists must critically evaluate the socio-political contexts of their clients, using the lens of political occupational therapy to integrate structural analyses into practice and ensure advocacy efforts effectively dismantle systemic injustices. Structural analyses in practice involve examining systemic inequities, such as policies restricting mobility, barriers to resource access, and censorship, to identify their impact on occupational participation. These analyses guide interventions by highlighting areas where advocacy and community support can be most effective, such as lobbying for policy changes to ensure equitable access to education and healthcare or supporting grassroots organizations in rebuilding community networks disrupted by governance shifts.

### 4.4 Interdisciplinary collaboration for systemic change

Occupational therapists should also pursue interdisciplinary collaborations with experts in sociology, law, political science, and human rights. These partnerships could generate actionable solutions, such as drafting policy recommendations grounded in occupational justice principles or mapping the societal impacts of governance measures. For example, joint efforts with legal advocates could help develop policies ensuring equitable access to public resources, while sociological studies might inform the design of culturally relevant interventions to restore community cohesion. Such collaborations should explicitly include occupational science professionals, whose theoretical expertise in the socio-political determinants of occupation can complement the applied, practice-oriented skills of occupational therapists. Joint OT–OS initiatives can strengthen evidence-based advocacy and intervention design in politically volatile contexts (Wilcock and Hocking, 2024).

### 4.5 Political activities of daily living in occupational therapy

Central to the advocacy work within political occupational therapy is the recognition of political Activities of Daily Living (pADL), a concept introduced by Kronenberg and Pollard (2005). pADLs encompass all advocacy activities that are inherently political in nature, including actions like lobbying, voting, protesting, or engaging in policy reform. These activities, though often categorized as “political,” are in fact integral to the daily lives of individuals and communities, particularly when political systems impose barriers that restrict access to fundamental rights and participation. In the context of martial law and governance shifts, pADLs become crucial tools for advocating for occupational justice, as they directly challenge systemic restrictions that hinder mobility, education, and social engagement. Understanding and engaging in pADLs allow occupational therapists to not only restore but also enhance their clients’ access to political participation and societal involvement, which are key components of occupational justice.

### 4.6 Advancing political occupational therapy education and practice

It is essential for occupational therapists to understand and actively engage in pADLs, as these actions directly align with the profession’s commitment to promoting autonomy, participation, and equity. During times of political instability or governance shifts, the ability of occupational therapists to advocate for clients and communities through pADLs is not just beneficial but imperative. This necessitates the inclusion of pADL education within occupational therapy curricula. By equipping students with the knowledge and skills to navigate and implement pADLs, educational programs can prepare future practitioners to respond proactively to the political and social challenges their clients may face. Training in policy analysis, advocacy strategies, and the intersection of politics and occupational justice would enable occupational therapists to integrate pADLs into their daily practice, ensuring that systemic injustices are addressed and that their clients’ occupational rights are upheld.

### 4.7 Strengths and limitations

This study has several limitations and strengths. One limitation is the lack of primary data, as it relies on an exploratory review rather than firsthand accounts from individuals affected by martial law, limiting the depth of lived experiences captured. Additionally, the context-specific focus on South Korea makes the findings less generalizable to other geopolitical contexts with different histories of authoritarian rule. There is also potential for interpretive bias, as the researcher’s South Korean identity and professional background in occupational therapy may influence the analysis, despite efforts to maintain objectivity. Strategies employed to minimize this interpretive bias included maintaining a reflexive research journal throughout the analysis, seeking peer debriefing from scholars outside South Korea, and triangulating sources from occupational science, political science, and human rights literature to ensure a balanced interpretation. Strategies to mitigate this bias included reflexive journaling during analysis and triangulation of sources across disciplines to reduce single-perspective interpretation. However, the study has notable strengths, including its interdisciplinary perspective, which integrates occupational justice, political occupational therapy, and historical analysis to offer a comprehensive understanding of the intersection of governance and occupational engagement. The study is also highly relevant to policy and practice, as it highlights the need for occupational therapists to engage in advocacy and consider sociopolitical determinants of occupation. Lastly, by drawing on past instances of martial law, particularly the Gwangju Uprising, the study provides historical insights, revealing recurring patterns of occupational disruption and reinforcing the need for occupational therapy to address systemic barriers to meaningful occupation.

### 4.8 Implications for future research and practice

Future research should further explore the intersection of political governance and occupational justice by conducting empirical studies that capture lived experiences of individuals affected by governance shifts. Qualitative research focusing on occupational disruption during political crises could provide deeper insights into the specific needs and challenges faced by vulnerable populations. Additionally, studies should examine the long-term effectiveness of interventions aimed at restoring occupational engagement post-crisis.

From a practice perspective, occupational therapists should adopt a proactive stance by integrating political occupational therapy principles into both clinical and community-based settings. Practitioners must develop advocacy skills and engage in interdisciplinary collaborations to drive systemic change. There is also a need to expand training and education on policy analysis, human rights, and political determinants of health within occupational therapy curricula. By embedding these components into practice and education, the profession can strengthen its role in promoting occupational justice and supporting individuals and communities affected by political instability.

### 4.9 Conclusion: the future of political occupational therapy

In summary, political Activities of Daily Living (pADL) offer occupational therapists a framework to understand and engage in the essential advocacy actions that can restore and preserve occupational justice. By incorporating pADLs into practice, therapists can help dismantle barriers to participation and ensure that advocacy becomes an embedded part of occupational therapy practice. This integration not only empowers clients but also reinforces the profession’s commitment to systemic change and social equity.

By adopting these critical and collaborative approaches, occupational therapy can expand its scope through political occupational therapy to address the root causes of occupational injustices, inspiring systemic change and fostering resilience at all levels of society. This prompts a critical reflection: What specific roles can occupational therapists play in preventing the erosion of occupational justice in the face of brief but impactful governance shifts like the December 2024 martial law in South Korea? Addressing this question requires a proactive stance, encouraging practitioners to consider both immediate disruptions and systemic advocacy strategies that ensure lasting occupational justice. Political occupational therapy directly aligns with the principles of occupational justice by prioritizing autonomy, equity, and participation as foundational elements of systemic advocacy (Kim, 2024a). Even short-term martial law impositions can leave lingering effects, such as a weakened sense of occupational autonomy, diminished trust in public institutions, and disrupted community participation, which occupational therapy must address through both preventive and restorative interventions. Additionally, there is a pressing need for enhanced education and training in policy, occupational justice, and advocacy within occupational therapy programs. This could include coursework on analyzing political systems, practical advocacy strategies, and integrating occupational justice principles into policy development. Such educational initiatives would prepare future occupational therapists to navigate and influence complex socio-political landscapes effectively.

## 5 Conclusion

The declaration of martial law in South Korea in December 2024 serves as a stark reminder of the profound impact of political systems on occupational justice, particularly in terms of autonomy, equity, and participation. The restrictions imposed during the brief period of martial law disrupted individuals’ ability to engage in meaningful occupations, limited equitable access to resources, and stifled opportunities for communal participation. These elements highlight the critical need for examining how political actions shape occupational environments and justice. This critical review highlights the need for occupational therapy to expand its scope to include socio-political dimensions, advocating for systemic change and equity. For example, during the 2019 protests in Hong Kong, community-based occupational therapy initiatives provided support through mental health workshops and advocacy training for local leaders, demonstrating the profession’s potential to address socio-political challenges effectively. Similarly, in South Africa’s post-apartheid era, occupational therapists worked within communities to rebuild social ties and foster economic resilience, showcasing the transformative impact of integrating socio-political dimensions into practice. By embracing frameworks like critical occupational therapy and liberation theory, the profession can address gaps in existing literature and practice, positioning itself as a catalyst for justice and empowerment in oppressive contexts. Political occupational therapy can be practically applied by identifying and dismantling structural barriers, such as restricted mobility and access to resources, which emerge under martial law. For instance, therapists can work with affected communities to establish mobile occupational clinics to maintain healthcare access during governance shifts. Liberation theory emphasizes collective empowerment, which could involve organizing community-driven programs to rebuild trust and foster resilience, such as mutual aid networks or advocacy training for local leaders. Future research should focus on developing strategies for occupational therapists to navigate political risks while advocating for justice, ensuring the profession remains relevant in addressing complex global challenges. Specific areas for exploration could include designing comprehensive advocacy training programs that equip therapists with the skills to engage in policy reform, as well as developing frameworks tailored to promoting occupational justice in politically volatile settings. Additionally, examining the long-term impacts of temporary governance shifts, such as martial law, on occupational participation could provide valuable insights for preventive and restorative interventions.
